# Differences in the Cognitive Skills of Bonobos and Chimpanzees

**DOI:** 10.1371/journal.pone.0012438

**Published:** 2010-08-27

**Authors:** Esther Herrmann, Brian Hare, Josep Call, Michael Tomasello

**Affiliations:** 1 Max Planck Institute for Evolutionary Anthropology, Leipzig, Germany; 2 Department of Evolutionary Anthropology and Center for Cognitive Neuroscience, Duke University, Durham, North Carolina, United States of America; Queen Mary University of London, United Kingdom

## Abstract

While bonobos and chimpanzees are both genetically and behaviorally very similar, they also differ in significant ways. Bonobos are more cautious and socially tolerant while chimpanzees are more dependent on extractive foraging, which requires tools. The similarities suggest the two species should be cognitively similar while the behavioral differences predict where the two species should differ cognitively. We compared both species on a wide range of cognitive problems testing their understanding of the physical and social world. Bonobos were more skilled at solving tasks related to theory of mind or an understanding of social causality, while chimpanzees were more skilled at tasks requiring the use of tools and an understanding of physical causality. These species differences support the role of ecological and socio-ecological pressures in shaping cognitive skills over relatively short periods of evolutionary time.

## Introduction

Chimpanzees and bonobos are humans' closest living relatives (the common ancestor of all three was around 6 million years ago), and are themselves very closely related (common ancestor around 2 million years ago). Despite their evolutionary closeness, the behavior of these two great ape species differs in important ways, and this might lead us to hypothesize that their cognitive skills differ correspondingly. For example, in their natural habitats, chimpanzees are extractive foragers who use many different types of tools to obtain food from challenging places, whereas bonobos rely on tools very little [1], [2]. This might suggest different skills of causal analysis, since using tools effectively requires an understanding of the physical requirements of the situation. On the other hand, bonobos are viewed as being more socially cooperative, and they are temperamentally shyer of new things and more tolerant of others in feeding situations [3], [4]. Given recent results with young children in which a clear connection between shy temperament and “theory of mind” skills has been demonstrated [5], we might expect bonobos to have better social-cognitive skills than chimpanzees.

In order to test these hypotheses, in the current study we looked for cognitive similarities and differences in juvenile and adult bonobos and chimpanzees using a broad spectrum of 16 cognitive tasks covering both physical and social cognition. We tested a large number of bonobos and we compared them to similarly aged chimpanzees. The battery of tasks included numerous items assessing a variety of cognitive skills in both the physical and social domains (Primate Cognition Test Battery: PCTB, [6], and see Table 1 and Methods S1; [7]).

**Table 1:** Mean performance across scales for females and males of both species and results of species, sex and age differences for the entire sample and matched sample size (bold) and only for entire sample size (non-bold).

Scale	Description	Chimpanzees		Bonobos		Species	Sex	Age
		Female	Male	Female	Male			
**Space** (4 tasks)	Locating or tracking a reward after location changes.	0.69	0.73	0.71	0.67	ns	ns	positive
**Quantities** (2 tasks)	Discriminating quantity.	0.67	0.68	0.64	0.66	ns	ns	ns
**Tools & Causality** (4 tasks)	Causal understanding including tool use.	0.66	0.65	0.57	0.48	**Ch>Bo**	ns	positive
**Social learning** (1 task)	Solving a simple but not obvious problem by observing a demonstrated solution.	0.16	0.10	0.13	0.05	ns	**m<f**	ns
**Communication** (3 tasks)	Understanding and producing communicative gestures.	0.58	0.56	0.59	0.46	ns	m<f	ns
**Theory of mind** (2 tasks)	Gaze following and understanding what an actor intended to do.	0.40	0.41	0.51	0.54	**Bo>Ch**	ns	negative

(Ch  =  Chimpanzee, Bo  =  Bonobo, f  =  female, m  =  male).

The range of cognitive tasks administered has been designed to test the two major evolutionary hypotheses regarding potential species differences in performance. Given high levels of genetic and ecological similarities [8], [9] as well as similar cognitive performance on a range of social and physical tasks (e.g. geometric gaze-following: [10]; gestural communication: [11], [12] quantitative discrimination: [13]; liquid conservation: [14] tool properties: [15]; motoric inhibitory control: [16]), phylogenetic inertia predicts few if any significant species differences between the two *Panin* species. In contrast, a socio-ecological model predicts that elements of the battery testing skills related to significant behavioral differences between the two species will reveal species differences in cognitive performance (i.e. even though relative to other apes these two species are highly genetically and ecologically similar). With their more cautious temperament [3], [17], social tolerance [4], [18], [19] and passive coping style [20] bonobos are more likely to outperform chimpanzees in theory of mind tasks, mirroring the positive relationship between shy temperament and theory of mind performance in young children [5]. However, given their greater dependence on a larger range of tools in the wild chimpanzees are likely to outperform bonobos on tasks relating to tool use and causality [2], [21].

## Methods

### Ethics Statement

The presented study was non-invasive and strictly adhered to the legal requirements of the countries in which it was conducted. The study was approved by an internal ethics committee at the Max Planck Institute for Evolutionary Anthropology. Animal husbandry and research complied with the “PASA Primate Veterinary Healthcare Manual” and the policies of Chimpanzee Sanctuary & Wildlife Conservation Trust, Uganda, Tchimpounga Chimpanzee Sanctuary, Republic of Congo and Lola ya Bonobo Sanctuary, Democratic Republic of Congo. The vast majority of chimpanzees and bonobos had access to large tracts of tropical forest (5–40 hectares) during the day. In the evening all apes came back from the forest and stayed the night in indoor enclosures (12 m^2^–160 m^2^). Apes voluntarily participated in the study and were never food deprived for any reason and they were fed, in addition to the food the apes could eat in the forest, a variety of fruits, vegetables, and other species-appropriate food two to four times daily. Water was either available ad libitum or was given to the subjects several times a day (since most of the apes at the sanctuary spent the day in the forest).

### Subjects

We tested 34 bonobos (21 males and 13 females; 5 to 22 years; mean age: 8.5) and 106 chimpanzees (53 males and 53 females; 3 to 21 years of age; mean age: 9.8 and mean age for subsample: 8.5). The bonobos lived at Lola ya Bonobo sanctuary, Democratic Republic of Congo. The chimpanzees lived either at the Ngamba Island chimpanzee sanctuary, Lake Victoria, Uganda, or at the Tchimpounga chimpanzee sanctuary, Republic of Congo. All apes came to the sanctuaries as orphans as a result of the illegal bushmeat trade, were raised by humans together with peers, and at the time of testing the majority lived in social groups.

The chimpanzee data was previously used in Herrmann et al. [6]. Rates of cognitive development differ in bonobos and chimpanzees, and in order to identify the differences in their cognitive skills we did not test infants and used certain methods to control for the effects of age on our sample. Because bonobos and chimpanzees differ in the development of their cognition we did not test infants and used methods to control for the effects of age on our sample in order to identify differences in the cognitive skills of the two species [18].

### Procedure

Subjects were tested on the PCTB that comprised 16 different physical and social cognitive tasks (see Table 1, Methods S1 and original study [6]). The tasks dealing with the physical world consisted of problems concerning *space* (4 tasks), *quantities* (2 tasks), *and tools and causality* (4 tasks). The scale space comprised tasks in which the ape had to either locate a reward (spatial memory), track a reward after invisible displacement (object permanence), after a rotation manipulation (rotation) or after location changes (transposition). The scale quantities was divided into problems in which the ape had to discriminate quantities (relative numbers) or had to discriminate quantities with added quantities (addition numbers). The causality scale included tasks in which the ape had to show a causal understanding either of noise produced by a hidden reward (noise), or a change in appearance produced by the hidden reward (shape) in addition to two tool use tasks. In the first task (tool use) the ape had to use a stick in order to retrieve a reward which was out of reach whereas in the second task the ape had to discriminate between a functional and a nonfunctional tool (tool properties). The tasks related to the social world consisted of problems requiring subjects to imitate another's solution to a problem (*social learning*, 1 task), communicate nonverbally with others (*communication*, 3 tasks), and understand goals and perceptions (“*theory of mind*”, 2 tasks). The scale communication comprised of one task in which the ape had to understand communicative cues indicating a reward's hidden location (comprehension) and two tasks in which subjects had to produce communicative gestures in order to retrieve a hidden reward (pointing cups and attentional state). The theory of mind scale was divided into a task in which the ape had to follow an experimenter's gaze to a target (gaze following) and a second in which the subject had to understand what an experimenter intended to do (intentions).

No individual had previously participated in a similar study and therefore all individuals were naïve to the test situation and tasks. Participants were tested individually by a human experimenter. Each participant completed all tasks in the PCTB within 3 to 5 hours, in the same order across several days of testing. For 11 tasks the subject had to make a choice between two or three potential hiding places. A human experimenter (E) sat behind a table facing the subject through a mesh panel or a Plexiglas window with three holes, through which subjects could insert a finger to indicate their choice. Different setups were used in 5 other tasks. Subjects either had to use a simple tool, solve a simple but not obvious problem by observing a demonstrated solution, gesture to the experimenter or follow the experimenter's gaze direction. The experimenter always waited until the subject was facing her before beginning a trial (more details on each task in Methods S1). For trials requiring a choice, the position of the reward was counterbalanced across either two or three locations, but the reward was never hidden for more than two consecutive trials in the same place.

### Coding and Data analyses

All testing was videotaped. Subjects' responses were initially coded live by E except for gaze-following trials, which E coded from videotape after the test. To be conservative, a reliability coder then independently scored (from videotape) 100% of the trials for chimpanzees. After excellent reliability was established for this species, a second coder then scored the standard 20% of the bonobo trials. The inter-observer agreement for all tasks combined was 99% for both species (for each scale see Table S1).

For the statistical analysis we calculated the proportions of correct responses for each scale. Six separate analyses of covariance (ANCOVA) were carried out, with species and sex as between-subject factors, performance on the six different scales as dependent variables and age as a covariate, to control for the influence of individual differences in age on the cognitive performance.

## Results

Table 1 presents comparisons based on the mean percentage of correct trials in each of the six cognitive scales administered as a function of species, sex and age. In support of the behavioral ecological model there were significant differences between species in only two out of six scales – both of which are consistent with observed species differences in behavior. Bonobos scored significantly higher on the “theory of mind” scale (F _1,135_ = 21.740, p<0.001) while chimpanzees outperformed bonobos in the tools and causality scale (F _1,135_ = 23.669, p<0.001). In this sample of juvenile and adults, age only had a significant influence in the scales where a species difference was found (theory of mind: F _1,135_ = 7.606, p = 0.007; tools and causality: F _1,135_ = 12.652, p = 0.001) with older individuals outperforming younger ones in the tools and causality scale (Pearson r = 0.327, p<0.001). The opposite was true in the theory of mind scale (Pearson r = −0.263, p = 0.002). In addition, there was a strong tendency for performance to improve with age in the space scale (F _1,135_ = 3.849, p = 0.052; Pearson r = 0.179, p = 0.034). Importantly, age effects did not explain the species differences in performance observed in the theory of mind and tools and causality scales, since no interactions between age and species were detected. Sex differences were also detected in two of the social scales. Females outperformed males in the communication tasks (F _1,135_ = 6.427, p = 0.012) and showed a strong tendency to outperform them in the social learning scales (F_1,135_ = 3.593, p = 0.06).

Due to the disparity in sample size between species, and given the potential effect of age on performance, we repeated the analysis after creating an even more conservative sample. Thus, we matched the sample size by selecting chimpanzees that best matched the age and sex composition of the bonobos (n = 34 for both species; 72 chimpanzees were dropped from this secondary analysis). This analysis replicates the species differences observed in both the tools and causality scale and the theory of mind scale (theory of mind: F_1,63_ = 9.962, p = 0.002, bonobos > chimpanzees; tools and causality: F_1,63_ = 15.891, p<0.001, chimpanzees > bonobos). In this analysis females again outperformed males in the communication scale (F _1,63_ = 4.823, p = 0.032) but not in the social learning scale (F _1,63_ = 1.187, p = 0.281) while previously detected age differences were no longer significant (p>0.064, in both cases).

## Discussion

Overall this broad spectrum comparison of bonobo and chimpanzee cognition demonstrates that species differences in cognition are directly reflected in the most pronounced differences observed in their naturally occurring behavior. Each species outperformed the other on one cognitive scale and in the direction predicted by previous socio-ecological observations, even when controlling for effects of age (i.e. statistically and matching ages). Mirroring individual differences observed in theory of mind development in human children [5], the more cautious and socially tolerant bonobo outperformed chimpanzees on the theory of mind scale. Meanwhile, the prolific tool-using chimpanzee, whose survival is more dependent on extractive foraging, outperformed bonobos in the tool-use and causality scale. This pattern can potentially be interpreted as suggesting that bonobos are more skilled at solving problems requiring an understanding of social causality, while chimpanzees are more skilled at solving problems relating to physical causality. In contrast, the two species did not differ in the scales measuring their understanding of problems related to spatial comprehension, discriminating quantities, using and comprehending communicative signals and learning from others via a social demonstration. This pattern of findings provides support for the hypothesis that socio-ecological pressures play an important role in shaping the cognitive differences observed between these species.

Our sample also allows us to consider differences in performance between sexes in a way that was not possible before. Interestingly, there was little difference between the sexes in their performance across the majority of tasks and the differences were largely inconsistent with previous observations. It is well established that male mammals including humans tend to outperform females on tasks relating to spatial rotation [22] but we did not see the same sex difference here in *Panins*. Moreover, there is little reason to suspect a sex difference between the communicative behavior of male and female *Panins* [12], yet in this study females outperformed males on tasks related to communication. The only sex difference that is consistent with previous behavioral observations is that of females outperforming males in the social learning scale (considering only the entire sample). Long-term observations of wild chimpanzees have suggested that female chimpanzees acquire more proficient tool-using techniques faster than males [23], [24] and other studies show a similar pattern in captive bonobos [25]. Therefore, it may be that socio-ecological pressures play a more limited role in producing cognitive differences based on sex in these species, but it also suggests that female *Panins* pay closer attention to others which allows them to learn and solve social problems more quickly and skillfully than males (while both sexes perform similarly in physical cognition tasks).

Finally, while we tried to control the effect of age in our comparisons across species and sex, age is also an important factor to consider in comparing these two species. Wobber et al [18] recently found that bonobos showed delayed development in behaviors and cognitive skills relating to feeding ecology. When we look at the overall effects of age in our analysis we do see that the *Panins* show developmental patterns, but they are somewhat inconsistent (i.e. when the two species are considered together: in some scales they improve in performance with age (space and tools & causality) while in another scale they show decreasing performance (theory of mind)). In general, it has been shown that inferential abilities in apes increase with age [26] and in particular in the wild older individuals outperform younger individuals in using tools [9], [27]. However, a decrease in performance in the theory of mind scale is surprising, but it is not unprecedented (mirror self recognition: [28]; episodic-like memory: [29]).

Overall, this study provides the first experimental comparison of our two closest living relatives in a wide range of cognitive tasks that allow us to examine both species and sex differences in cognitive performance (see [30] for a related primate-wide meta-analysis). While the performance of the two species was mostly similar, the cognitive performance of the two species differed in ways that are consistent with the most pronounced differences observed in their natural behavior. In other words, while the two species are highly similar and only diverged 1–2 million years ago, the observed socio-ecological differences may have shaped each species psychology in predictable ways. The close genetic relationship between chimpanzees and bonobos and the release of the bonobo genome will permit future comparisons between the genomes of the two species which should aid in identifying heritable differences that underlie any such cognitive differences. Understanding how development evolved between bonobos and chimpanzees can then inform hypotheses regarding cognitive evolution in our own species from our last common ancestor with the *Panins*.

## Supporting Information

Methods S1 Methods for the Primate Cognition Test Battery.
(0.09 MB DOC)

Table S1 Inter-observer reliability.
(0.03 MB DOC)
