# Supplementary material for: Differences in the Cognitive Skills of Bonobos and Chimpanzees
Source: PLoS One. 2010 Aug 27;5(8):e12438. doi: 10.1371/journal.pone.0012438 (PMC2929188; doi:10.1371/journal.pone.0012438)
Supplement: Methods S1 — Methods for the Primate Cognition Test Battery. (0.09 MB DOC) [file pone.0012438.s001.doc]

**Supporting Information for:**

**Differences in the cognitive skills of bonobos and chimpanzees**

Esther Herrmann, Brian Hare, Josep Call, and Michael Tomasello

**Methods for the Primate Cognition Test Battery (taken from supplementary online material in Herrmann et al. 2007)**

***Materials and Apparatus***

A wooden table (80 cm × 39 cm) with a sliding platform (78 cm × 35 cm) was used for the experiment. Three blue (Ø 10 cm × 8.3 cm height) or other materials (which are reported in Procedures section) were used to cover / present the food reward; these were placed on the sliding platform 58 cm apart (in case of three cups ~20 cm apart). The testing table on which the sliding platform was attached was either placed in front of a Plexiglas testing window (69 cm × 48 cm) with three hand holes (6 cm in diameter) or a mesh panel (opening in the mesh 5 cm^2^). Subjects could reach their fingers through the holes or the mesh wire to make a choice. Throughout testing, unless otherwise indicated, a choice was scored as the first cup that a subject indicated with their finger. Different cups and containers were used as hiding places. When the apes indicated the correct location they were given a small food reward. However, unless otherwise stated, when the apes made incorrect responses they were always shown the location of the hidden food after each trial. The same desirable food items were used as rewards for most of the tasks. For chimpanzees, this consisted mainly of banana pieces, whereas apple pieces served as rewards for bonobos. However, depending on the subjects’ motivation and some of the demands of the different tasks, different food rewards were used (i.e. for the quantity tasks, either raisins or peanuts were used as countable food items).

***Procedures***

**Physical Domain**

**1. Space**

**a. Spatial Memory**

The experimenter (E1) showed the subject two rewards and then placed the rewards under two of the three cups, which stood in a row on the platform while the subject was watching. E1 then pushed the platform forward and the subject was allowed to make up to two choices in succession. However, if subjects chose the empty container, no further choices were possible. To count as a correct response subjects had to choose both baited cups in succession.

**b. Object Permanence**

The experimenter (E1) placed three empty cups in a row on the platform. Then E1 placed an additional small opaque cup on the far left side of the platform and placed a reward under it in full view of the subject. After the reward was placed under the small cup, one of three possible displacements was conducted:

Single displacement: The experimenter moved the small cup containing the reward under one of the three cups, leaving the reward under this cup without touching the other two cups.

Double adjacent displacement: The experimenter moved the small cup containing the reward under two adjacent cups in succession, leaving the reward under one of these cups without touching the third cup.

Double non-adjacent displacement: The experimenter moved the small cup containing the reward under the left and right cups while skipping the center cup (i.e. not touching it). As before, the reward was left under one of the two manipulated cups.

After hiding the food piece under one of the cups, E1 showed the subject that the small cup, used to bait the other cups, was empty. Then the subject was allowed to make either one choice (in the single displacement item) or up to two choices (in the double displacement items). If the subject chose a cup that had not been manipulated during the demonstration, no further choices were possible. To count as a correct response subjects had to choose the baited cup before choosing a cup which was not manipulated at all.

**c. Rotation**

Three cups were aligned in a row on a tray placed on the testing platform. E1 then showed a reward to the subject and placed it (in view of the subject) under one of the three cups. Then the tray was spatially manipulated in one of three ways and the subject was allowed to choose one cup in order to correctly locate the food:

180° middle: The reward was placed under the middle cup, and the tray was

rotated 180° in clockwise direction. Thus, the reward was ultimately in the same location that it was originally placed.

360°: The reward was placed under either the left or right cup, and the tray was

rotated 360° in clockwise direction. After the rotation, the reward was in the same

location as it was originally placed.

180° side**:** The reward was placed under either the left or right cup, and the tray

was rotated 180° in clockwise direction. Therefore the reward was ultimately located on the opposite side of where it was originally placed.

To count as a correct response subjects had to choose the baited cup first.

**d. Transposition**

Three cups were placed in a row on the testing platform. E1 then showed a reward to the subject and placed it (in view of the subject) in one of the three cups. Then one of three manipulations of the baited cup was conducted and the subject was allowed to choose one cup:

Single transposition: E1 switched the position of the baited cup with one of the

empty cups without touching the third cup.

Double unbaited transposition: E1 first switched the baited cup with one of the

empty cups, and then switched the positions of the two empty cups.

Double baited transposition: E1 switched the baited cup with one of the empty

cups, and then switched the baited cup again with one of the empty cups.

To count as a correct response subjects had to choose the baited cup first.

**2. Quantities**

**a. Relative Numbers**

E1 placed two dishes on the testing platform, then hid them from the subjects’ view with an occluder. E1 then baited them with different amounts of equal sized food pieces (half a peanut or raisin was used as a unit). E1 then covered the dishes with lids and placed them in the middle on the platform. The occluder was then removed, after which E1 lifted the lids of both dishes simultaneously so that subjects could see the amounts in each. After ~5 seconds had passed, E1 moved the open dishes to the sides of the platform, one on the right and one on the left, and the subject was allowed to choose one. Each subject received one trial for each of the following pairs of numbers that were always presented in the same order for all subjects: 1:0, 5:1, 6:3, 6:2, 6:4, 4:3, 3:2, 2:1, 4:1, 4:2, 5:2, 3:1 and 5:3.

To count as a correct response subjects had to choose the larger quantity first.

**b. Addition Numbers**

E1 baited three dishes with different amounts of reward (same as in Relative

Numbers) behind an occluder, covered them with lids, and placed them in the middle of

the platform. The occluder was removed, and E1 then lifted the lids of the two side dishes simultaneously. After ~5 seconds, E1 covered the side dishes and uncovered the one in the middle. Subjects could view the contents of the center dish for ~5 seconds, and then E1 transferred the rewards from the center dish to one of the side dishes. During the transfer the subject could not see the content of the side dishes. Then E1 removed the empty center dish and the subject was allowed to choose between the two closed side dishes. Each subject received one trial for each of the following pairs in the same order: 1:0 + 3:0 = 4:0; 6:1 + 0:2 = 6:3, 2:1 + 2:0 = 4:1, 4:3 + 2:0 = 6:3, 4:0 + 0:1 = 4:1, 2:1 + 0:2 = 2:3, 4:3 + 0:2 = 4:5.

To count as a correct response subjects had to choose the larger quantity first.

**3. Causality**

**a. Noise**

The experimenter (E1) hid a reward in one of two opaque cups; during baiting the cups were behind an occluder so that the subjects could not see in which cup the reward was hidden. After removing the occluder, E1 manipulated the cups in one of two ways:

Noise full: The experimenter shook the baited cup three times so that the food

rattled inside and then simply lifted the empty one without shaking it.

Noise empty: The experimenter shook the empty cup (producing no sound) three times and then lifted the baited one without shaking it.

To count as a correct response subjects had to choose the baited cup first.

**b. Shape**

E1 hid a reward underneath one of two identical pieces of plastic or cloth, which

were placed on the platform. During baiting the pieces of material were behind an

occluder, so the subject could not see the experimenter placing the reward. Once the

occluder was removed, the subjects were confronted with one of two situations and were allowed to touch one piece of material to locate the hidden food:

Board: The reward was hidden underneath one of two plastic boards (20 x

15 cm). The food’s location was visually apparent since the baited board was inclined as it was placed on the food (the other board remained flat on the table).

Cloth: The food was hidden underneath one of two pieces of cloth (20 x 15 cm). The reward made a visible bump under this piece of cloth (the other cloth remained flat on the table).

To count as a correct response subjects had to choose the baited board or cloth first.

**d. Tool Properties**

E1 placed two different tools and an equal sized reward for each tool on the

platform behind an opaque screen. One tool was functional and could be used to retrieve the reward associated with it, whereas the second tool was non-functional and could not be used to obtain the associated reward. Each subject participated in five different items:

Side: The potential tools consisted of two identical pieces of cloth (20 cm x 15 cm). A reward was placed on top of one cloth piece, whereas the other reward was placed directly next to the other cloth piece (i.e. making the second tool ineffective for retrieving the food). Thus, the subject could only retrieve a reward by pulling the piece of cloth with the reward on top of it.

Bridge: A Plexiglas bridge was placed over each of the far ends of the two

identical cloth pieces. For one of the potential cloth tools, the reward was placed on top

of the bridge (making the tool ineffective in retrieving the food). On the other side, the

reward was placed on the cloth underneath the bridge. Thus, the subject could only obtain a reward by pulling the cloth with the reward placed directly on it.

Ripped: A rectangular, intact cloth piece (20 cm x 15 cm) was positioned on one side of the table, and two smaller cloth pieces (11 cm x 15 cm and 8 cm x 15 cm) were positioned on the other side. The two small pieces of cloth were placed on the table such that there was a 1 cm gap between them, which visually emphasized that they were disconnected. Importantly, when combined, the two small pieces of cloth – including the 1 cm gap between them – were of identical length and width as the one functional large piece. Once the cloths were in place, a reward was placed on top of the far end of the intact cloth, and the other was placed on the out of reach piece of the two disconnected pieces (making them ineffective as tools). The subject could only acquire a reward by pulling the large, intact cloth piece.

Broken wool: This item was identical to the Ripped cloth item with the exception that pieces of wool string were used instead of cloth: one piece of wool was intact, whereas the other was cut in two pieces. The reward was tied to the far end of the wool pieces out of the subject’s reach, so a reward could only be acquired by pulling the intact piece of wool.

Tray circle**:** Two small plastic trays (15 cm x 4.5 cm) were placed on the platform. One tray had a hole cut out of it that formed a circle (3.5 cm in diameter) around a reward placed inside. The other tray had a u-shaped hole cut out of the back of it so that if a reward was placed “in” it the tray surrounded the food but did not hold it. A string was then attached to both trays that could be used to pull the tray and the reward within reach. Subjects could obtain the reward if they pulled the rope which was attached to the tray with the circle-shaped hole, as the material at the back of the tray pushed the reward forward.

A correct response was scored if the subject first chose the functional tool by pulling it.

**Social Domain**

**1. Social Learning**

For each of the following social learning tasks the subject was given two minutes to solve the problem after E1 demonstrated the solution. For all three tasks we established a baseline with twelve chimpanzees during the pilot phase at the Wolfgang Köhler Primate Research Center in Leipzig, Germany. Without demonstration none of the twelve subjects solved the problem with the same means we demonstrated in the following three tasks (i.e. making it likely that any reproduction of the demonstrations described below are due to social influences).

A correct response was scored only if the subject not only obtained the reward but did so by using a means highly similar to that they had seen previously demonstrated.

**a. Paper Tube**

A reward was placed inside a 20 cm long transparent plastic tube with a piece of

paper attached over both ends. E1 demonstrated for the subject how to open the tube:

First E1 poked her finger through the paper on one end and then wiggled her finger in the tube to rip the paper further, making the hole in the paper larger (i.e. as opposed to using her mouth or hands to tear the paper off the tube). Finally, E1 tilted the tube in order to let the reward fall in her hand. After the demonstration E1 handed an identical tube to the subject.

**b. Banana**

A small slice of banana was placed in the center of a 30 cm long transparent Plexiglas tube. The reward was trapped in the tube, such that a specific force had to be applied to get the reward out of the tube. E1 demonstrated for the subject how to get the reward out by banging one end of the Plexiglas tube on the floor (as opposed to shaking it forcefully). After the successful demonstration, E1 handed an identical tube with a banana inside to the subject.

**c. Stick Tube**

An opaque plastic tube with caps on each end was baited with a reward. One of

the caps had a hole in it but was glued to the tube, whereas the other cap had no hole but could be removed. E1 demonstrated for the subject how to open the tube: First E1

inserted a stick through the cap with a hole, and then she pushed the stick through the

hole which forced the cap on the other end to fall off. After the successful demonstration E1 handed an identical grey tube to the subject.

**2. Communication**

**a. Comprehension**

The experimenter (E1) hid a reward under one of two cups that were placed in a

row on the testing platform behind an occluder. After removing the occluder, E1 gave

one of three social cues before giving the subject the opportunity to search in one of the

cups.

Look: E1 sat in front of the cup and alternated her gaze between the subject and

the baited cup three times while calling the subject’s name. After the first three gaze

alternations E1 continuously looked towards the cup until the subject chose.

Point: E1 sat in front of the cups and continuously pointed to the baited cup with

the extended index finger of her cross-lateral hand. At the beginning of the point, she

alternated her gaze between the subject and the cup three times while calling the subject’s name and then only stared in the baited cup’s direction.

Marker: E1 placed an iconic photo marker, which depicted the reward, on top of

the baited cup. Before the placement, E1 held the photo in her hand and alternated her

gaze three times between the photo and the subject while calling the subject’s name.

To count as a correct response subjects had to choose the baited cup first.

**b. Production: Point Cups**

In the following task, E1 indicates the person who was the main

experimenter throughout testing, whereas E2 indicates a second helper. Two cups served as hiding places. These cups were placed on two separate tables. The hiding places were spread 120 cm apart and both equidistant from the subjects’ starting point between the two hiding places. E2 entered the testing area, placed a reward under one of the two cups in full view of the subject, and then left the area. Then E1 entered the testing area and centered the ape by giving her a piece of food between the two tables. Then E1 stood equidistant to both cups and waited until the subject approached one cup and indicated it. If the subject indicated first the correct cup within one minute, E1 gave the reward to the subject and a correct response was scored.

**c. Production: Attentional State**

In the following task E1 indicates the person who was the main experimenter

throughout testing, whereas E2 indicates a second helper. Subjects participated in four

different items in which the attentional state of E1 varied. In all items the trial started

when E2 entered the testing area and placed a reward out of reach but in front of the

subject’s room – either on its right or left side. Then E2 left the area. Next E1 entered the testing area, but stood on the end of the room opposite of the reward and thus did not notice the reward on the floor. Then E1 stood and looked in four different ways:

Away: E1 turned around and looked away from the reward. The subject had to

approach E1 from her front in order to see each other. If the subject approached E1

within 20 seconds (20s), E1 turned around and waited for the subject to direct her

attention to the reward. If the subject went back to the reward’s location and indicated the reward within 20s, E1 handed the reward to the subject.

Towards: E1 looked towards the reward. E1 waited for the subject to approach the

reward and to direct her attention towards the reward within 20s. If the subject indicated the reward within 20s, E1 handed it over to the subject.

Away Body-facing: Identical to Away, except that E1‘s body faced toward the

reward and only the face was turned away.

Towards Body-away: Identical to Towards, except that E1‘s body was turned

away and only the face was directed towards the reward.

The subjects could indicate the location of the hidden food by pointing to the location of where the reward was hidden if the location was in view of E1 or by first moving into E1’s view (i.e. trying to obtain her attention) and then pointing to the correct location when E1 initially could not see the reward location. In all of the items, if the subject never approached or overtly indicated to E1 the location of the food, E1 left the testing area and E2 came back to remove the reward.

**3. Theory of Mind**

**a. Gaze Following**

E1 sat in front of the subject and gave a piece of food to the subject in order to attract her attention. Once the subject sat and looked at the experimenter, E1 started the trial. Each individual received three different items (which were conducted on a different day within the test battery to minimize any kind of habituation):

Head + Eyes**:** E1 called the subject’s name and showed them a piece of food before hiding it in her hand, which remained in front of her body. Then E1 looked up with both her head and eyes (eyes open) for ~10s.

Back: E1 sat with her back facing the subject. E1 called the subject’s name and

showed them a piece of food before hiding it in her hand, which remained next to her shoulder. Then E1 looked up to the ceiling for ~10s. Within the ~10s E1 looked back over her shoulder at the subject three times to ensure that the subject was still paying attention. If the subject was not paying attention when E1 looked the second time, the trial was repeated.

Eyes: E1 called the subject’s name and showed them a piece of food before hiding it in her hand, which remained in front of her body. E1 glanced up at the ceiling for ~10s while her face was still facing the subject.

A correct response was obtained if the subject followed the gaze of the experimenter.

**b. Intentions**

E1 hid a reward in one of two cups placed in a row on the platform behind an

occluder. After removing the occluder, E2 manipulated the cups in one of two ways:

Trying: E2 tried unsuccessfully to open the baited cup by removing the lid while

looking at the cup.

Reaching: A mesh or Plexiglas barrier blocked E2’s access to the cups. E2

unsuccessfully tried to reach the baited cup by extending the equilateral arm, looking at

the correct cup. She continued to give this cue until the subject indicated a choice.

For both items, ~3s after each demonstration E1 approached the table and pushed the platform forward so that the subject was allowed to make a choice. To count as a correct response subjects had to choose the baited cup first.
