# Supplementary material for: Differences in the Cognitive Skills of Bonobos and Chimpanzees
Source: PLoS One. 2010 Aug 27;5(8):e12438. doi: 10.1371/journal.pone.0012438 (PMC2929188; doi:10.1371/journal.pone.0012438)
Supplement: Table S1 — Inter-observer reliability. (0.03 MB DOC) [file pone.0012438.s002.doc]

**Supporting Information for:**

**Differences in the cognitive skills of bonobos and chimpanzees**

Esther Herrmann, Brian Hare, Josep Call, and Michael Tomasello

**Reliability**

**Table S1** Inter-observer reliability for the whole PCTB and by scale and species

|  | Agreement of first and second observer (percent) | |
| --- | --- | --- |
|  | Chimpanzee | Bonobo |
| **Overall PCTB** | 99.0 | 99.3 |
| Space | 99.9 | 98.8 |
| Quantities | 99.7 | 100.0 |
| Tools & Causality | 99.4 | 100.0 |
| Social Learning | 99.4 | 100.0 |
| Communication | 99.4 | 99.4 |
| Theory of Mind | 96.2 | 97.6 |
